# Correction to “Circ‐MALAT1 accelerates cell proliferation and epithelial mesenchymal transformation of colorectal cancer through regulating miR‐506‐3p/KAT6B axis”

**DOI:** 10.1002/kjm2.12846

**Published:** 2024-05-10

Yang F‐S , Gong S‐X , Qiu D‐D . Circ‐MALAT1 accelerates cell proliferation and epithelial mesenchymal transformation of colorectal cancer through regulating miR‐506‐3p/KAT6B axis. Kaohsiung J Med Sci. 2023;39(9):862–872. 10.1002/kjm2.12698 37272875 PMC11895866

In this article, the following funding information should have been included.

Scientific Research Project of Hunan Provincial Health Commission, Grant/Award Number: 202204013155

We apologize for this error.

